# Evaluation and Comparison of Multiple Test Methods, Including Real-time PCR, for *Legionella* Detection in Clinical Specimens

**DOI:** 10.3389/fpubh.2016.00175

**Published:** 2016-08-31

**Authors:** Adriana Peci, Anne-Luise Winter, Jonathan B. Gubbay

**Affiliations:** ^1^Public Health Ontario, Toronto, ON, Canada; ^2^University of Toronto, Toronto, ON, Canada

**Keywords:** *Legionella*, PCR, culture, urine antigen, BAL, sputum

## Abstract

*Legionella* is a Gram-negative bacterium that can cause Pontiac fever, a mild upper respiratory infection and Legionnaire’s disease, a more severe illness. We aimed to compare the performance of urine antigen, culture, and polymerase chain reaction (PCR) test methods and to determine if sputum is an acceptable alternative to the use of more invasive bronchoalveolar lavage (BAL). Data for this study included specimens tested for *Legionella* at Public Health Ontario Laboratories from 1st January, 2010 to 30th April, 2014, as part of routine clinical testing. We found sensitivity of urinary antigen test (UAT) compared to culture to be 87%, specificity 94.7%, positive predictive value (PPV) 63.8%, and negative predictive value (NPV) 98.5%. Sensitivity of UAT compared to PCR was 74.7%, specificity 98.3%, PPV 77.7%, and NPV 98.1%. Out of 146 patients who had a *Legionella-*positive result by PCR, only 66 (45.2%) also had a positive result by culture. Sensitivity for culture was the same using either sputum or BAL (13.6%); sensitivity for PCR was 10.3% for sputum and 12.8% for BAL. Both sputum and BAL yield similar results regardless testing methods (Fisher Exact *p*-values = 1.0, for each test). In summary, all test methods have inherent weaknesses in identifying *Legionella*; therefore, more than one testing method should be used. Obtaining a single specimen type from patients with pneumonia limits the ability to diagnose *Legionella*, particularly when urine is the specimen type submitted. Given ease of collection and similar sensitivity to BAL, clinicians are encouraged to submit sputum in addition to urine when BAL submission is not practical from patients being tested for *Legionella*.

## Introduction

*Legionella* is a Gram-negative, rod-shaped bacterium of the *Legionellaceae* family that exists ubiquitously in soil and water (1, 2). There are more than 50 *Legionella* species and 70 serogroups currently. Of all *Legionella* species, 21 species are believed to cause human disease (2). *Legionella* can cause two distinct clinical presentations: Pontiac fever (PF), a mild upper respiratory infection with non-specific influenza-like illness symptoms and Legionnaire’s disease (LD), which has a more severe clinical presentation including pneumonia (3). Both PF and LD are underdiagnosed; patients with PF have mild, self-limiting influenza-like symptoms that mimic those of other respiratory pathogens. Individuals with LD are often diagnosed with community-acquired pneumonia, treated empirically, and do not receive laboratory testing. In addition, because of ease of collection, clinicians often use urine specimens to test for *Legionella* using commercial urinary antigen tests (UAT), most of which only detect *Legionella pneumophila* serogroup 1. Testing of urine for *Legionella* antigen has a reported sensitivity of 70–80% when compared to *Legionella* culture of respiratory specimens and does not allow for linkage of human isolates with *Legionella*-positive environmental samples, which is usually done using sequence-based typing (SBT) (4). In a recent *Legionella* study, more patients were confirmed for *Legionella* by UAT than culture or polymerase chain reaction (PCR); the authors hypothesized that the increased sensitivity compared to culture was because most cases were community acquired with less severe disease and the increased sensitivity over PCR was due to the predominance of *L. pneumophila* serogroup 1 (5).

Culture-based testing can help to identify links between environmental sources and human isolates and has traditionally been considered as the gold standard for diagnosis of *Legionella* (2). In Ontario, the primary specimen type to undergo culture testing has historically been obtained through bronchoalveolar lavage (BAL), a procedure that is only used for a minority of patients tested for *Legionella* because of its invasive nature. Testing of sputum from the upper respiratory tract through culture-based methods is potentially a viable non-invasive alternative; however, reports from other studies have indicated variable sensitivity from less than 10 to 80%, and its use is often limited by the fact that many patients with LD do not produce sputum (6).

Public Health Ontario Laboratories (PHOL), which performs the majority of testing for *Legionella* in the province of Ontario, Canada has historically used UAT and/or culture of respiratory specimens, most commonly BAL specimens, in order to detect *Legionella* in patients with suspected infection due to this pathogen. PCR for detection of *Legionella* in clinical samples was introduced at PHOL on 28th May, 2012, using a protocol developed at the Center for Disease Control and Prevention, USA (US CDC) (7). PCR is a sensitive and rapid test that can detect *Legionella* species from clinical and environmental sources. Similar to culture, PCR is able to detect all *Legionella* species and serogroups. Testing of specimens early during the course of disease and obtaining specimens from the lower respiratory tract has been reported to result in improved PCR sensitivity (8). Sensitivity of PCR compared to culture was reported to be 100% (9). In another study, PCR was reported to be better than culture at detecting less severe disease (10). When molecular typing is indicated, SBT can often be performed on primary *L. pneumophila* PCR-positive specimens, which are culture-negative (based on PHOL experience). Avni et al. compared results of PCR in respiratory specimens with UAT results and reported a better sensitivity of PCR than UAT and similar specificity (11). PCR findings led to reclassification of 18% of LD symptomatic cases with previously negative UAT.

Comparing the performance of different test methods is challenging since there is an absence of a true gold standard method; the commonly used BinaxNOW^®^ UAT only identifies *L. pneumophila* serogroup 1 while culture has been reported to have a low sensitivity. In addition, PCR may result in false-positive results due to contamination of test reagents with *Legionella* DNA (12).

The purpose of our study was to describe *Legionella* testing at PHOL, to compare the performance of urine antigen, culture and PCR test methods, and to determine if sputum is a viable specimen alternative to the use of more invasive BAL for *Legionella* PCR and culture, especially for investigations when there is a need to link human isolates to environmental sources.

## Materials and Methods

Data for this study included specimens tested for *Legionella* at PHOL from 1st January, 2010 to 30th April, 2014. Specimens included in this study were tested as part of routine clinical testing.

### Test Methods

PHOL tested urine specimens for *L. pneumophila* serogroup 1 using Alere BinaxNOW^®^ *Legionella* Urinary Antigen Card (MA, USA), and all appropriate specimens such as aspirate, BAL, lung tissue, sputum, nasopharyngeal swab, pleural fluid, etc., were tested for *Legionella* by culture until 28th May, 2012. After this date, PHOL implemented PCR testing for *Legionella* on respiratory specimens, and culture was only used on specimens testing positive by PCR for the purpose of facilitating molecular typing by SBT when indicated.

Culture-based testing for *Legionella* was performed using buffered charcoal yeast extract-based agar (BCYE) and buffered polymyxine B, anisomycin, and vancomycin (BPAV) charcoal media. The plates were re-incubated at 35°C for up to 14 days and examined under a microscope daily for colonial morphology such as a mottled surface and iridescent red–blue–green sheen or a faceted cut-glass appearance. *Legionella* growth was visible only after at least 3 days of incubation. All non-urine specimens tested for the presence of *Legionella* DNA were examined using real-time PCR with primers and probes as described in US CDC’s published methodology (7). The PCR has two probes, one for all *Legionella* species, and one for *L. pneumophila*. Primers targeting the 23S-5S rRNA intergenetic spacer region conserved for all *Legionella* species were used, as previously published by US CDC (7). The forward and reverse primers were 5′-GTA CTA ATT GGC TGA TTG TCT TGA CC-3′ and 5′-CCT GGC GAT GAC CTA CTT TCG-3′, respectively. Two probes were designed within the amplicon region. One is specific for *L. pneumophila* (5′-Cal Flour Orange 560-ATC GTG TAA ACT CTG ACT CTT TAC CAA ACC TGT GG-3′BHQ), the other one recognizes all known *Legionella* species (5′-FAM ATC TC“G” AA“C” T“C”A “G”AA “G”T“G” AAA C-3′BHQ) (“” denotes locked nucleic acid), and is referred to as the genus-wide probe or *Legionella* spp. probe.

Slide agglutination testing (SAT) was used to speciate and serogroup *L. pneumophila* isolates and atypical *Legionella*-like isolates. Known antibodies were mixed with culture isolates on a glass microscope slide. Antibodies against 50 *Legionella* species/serogroup targets were used for this method based on the protocol developed by US CDC (13). Agglutination reactions were scored on a scale from 4 + (strongest) to 1 + (barely visible). In cases of cross-reaction (two or more species/serogroup targets reactive), results were compared against specific positive and negative controls. The strongest reaction was considered the final result.

### Data Transformation

First, the data were converted from the test to specimen level as more than one test may have been performed per specimen. In the event of discrepant results between different test methods, a positive result took precedence over an indeterminate or a negative result. In the event of more than one positive result for a single specimen, the result that reflected a more detailed level of identification was considered the final outcome; for example serogroup took precedence over species. Second, the data were transformed from the specimen to patient level, as more than one specimen may have been tested per patient. Patients were identified based on their health card number or first name, last name, and date of birth if the health card number information was missing. Third, separate patient episodes were identified as some patients were tested for *Legionella* more than once a year or during the study period; we determined each unique episode to span 90 days from the time the first specimen was received. Specimens were considered to be part of a single episode if the period between the first and subsequent specimen submission dates was ≤90 days. The time was reset for the next consecutive 90-day timeframe for specimens from the same patient received after 90 days. When there was more than one discrepant result per patient episode, a final result was assigned for each patient with a positive result taking precedence over an indeterminate or a negative result. In addition, a *Legionella-*positive specimen identified to a more detailed level took precedence over any other *Legionella-*positive specimens, and the earliest date was used to identify that episode.

Another transformation was done at the test level to compare the performance of different specimen types and *Legionella* test methods. For both comparisons specimens submitted from the same patient episode within a 14 day lagging period from receipt of the first specimen were selected to avoid bias related to delays in specimen submission. For those patients for whom both sputum and BAL specimens were submitted, test results for each source were compared. Indeterminate results were excluded from these analyses.

For the purpose of this study, cases refer to individuals who tested positive for *Legionella* during a single episode, and controls are individuals who tested negative during a single episode. Patients refer to both cases and controls being tested for *Legionella* infection.

### Data Analyses

Statistical analyses were performed at the patient and specimen level using Stata/SE version 10.0 (StataCorp LP, College Station, TX, USA). Data for this study were analyzed two ways: by patient episode and by test method. Patient episode data were used to describe the patients’ demographic characteristics and to describe the seasonality of *Legionella* including trends. Data for 2014 were not complete and thus were not considered for the trend analysis. Chi-square test was used to compare demographic information between patients.

Specimen source and test method comparisons were performed at the test level. Sensitivity and specificity were reported, including 95% confidence intervals. Chi-square was used to compare sensitivity and specificity of test methods during different episodes. Fisher’s exact test was used in the event of a sample size less than five.

### Ethics

These data are also used for routine laboratory surveillance, which is a mandate of Public Health Ontario. Therefore, consultation with our organization’s privacy office or ethics committee was not required. To protect patient privacy and confidentiality, data are reported in an aggregated anonymized format.

## Results

**Figure 1:**
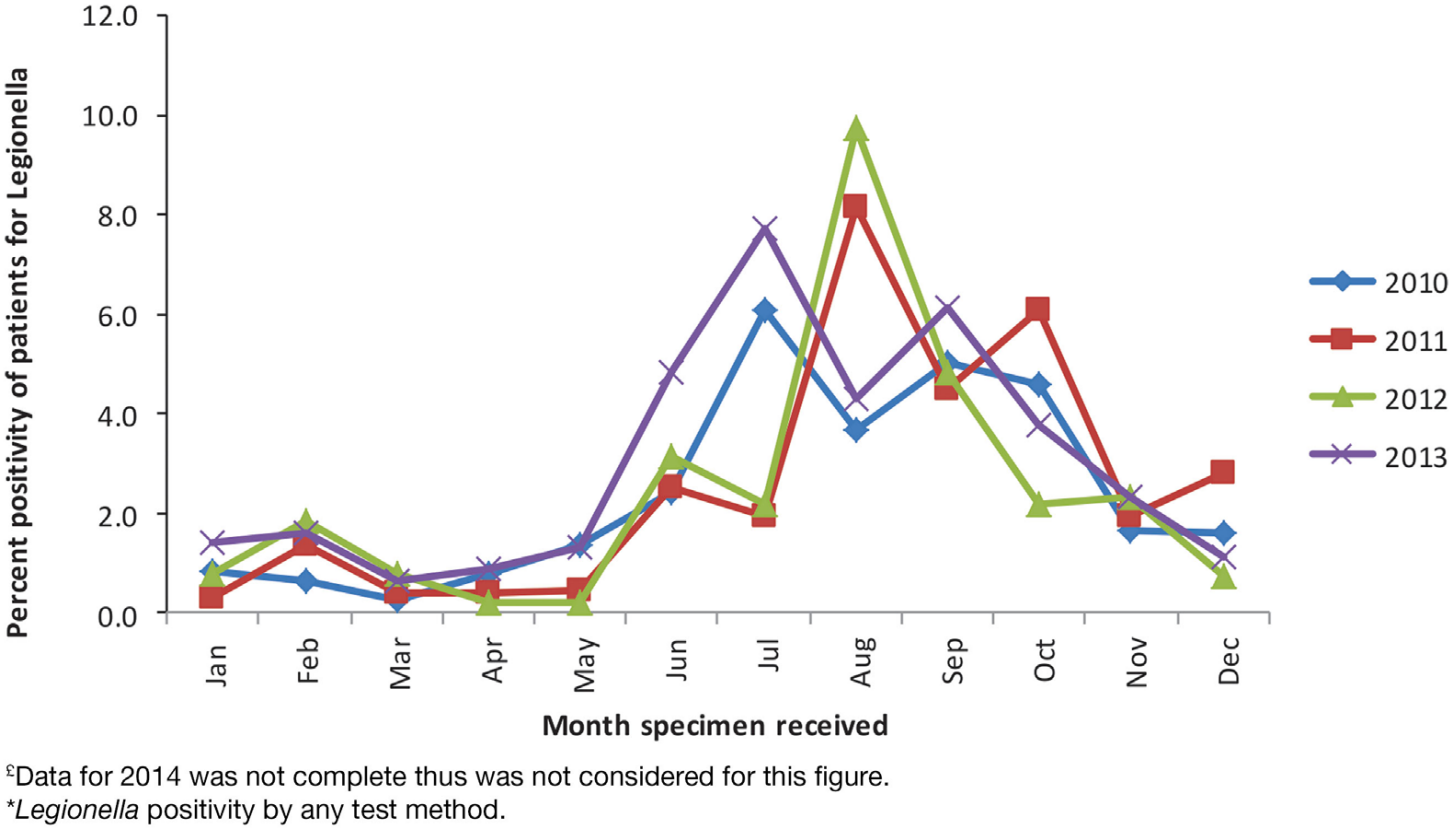
Patient percent positivity for *Legionella* by month, PHOL, 1st January, 2010 to 31st December, 2013^£^*.

During the study period, 28,965 patients were tested for *Legionella*. UAT was the main test method and was used for 23,389 (81%) patients. Culture-based testing was used for 3,832 (13%) patients and PCR for 3,661 (13%) patients. *Legionella* was detected by any test method in 725 (2.5%) patients. Percent positivity for UAT was 2.8% (649/23,389 patients tested), for culture was 2.8% (109/3,832), and for PCR was 3% (108/3,661). These results are not mutually exclusive because 2,083 (7.2%) patients were tested by more than one method, with UAT and PCR being the most common co-test methods, used in 1,048/28,965 (3.6%) patients. Since its implementation, PCR was used to diagnose *Legionella* among 23% of the patients. Peak detections occurred in July in 2010 and 2013 (6.1 and 7.7% of all patients tested for *Legionella* during that month, respectively) and in August in 2011 and 2012 (8.2 and 9.7%, respectively) (Figure 1).

### Patient Characteristics

**Figure 2:**
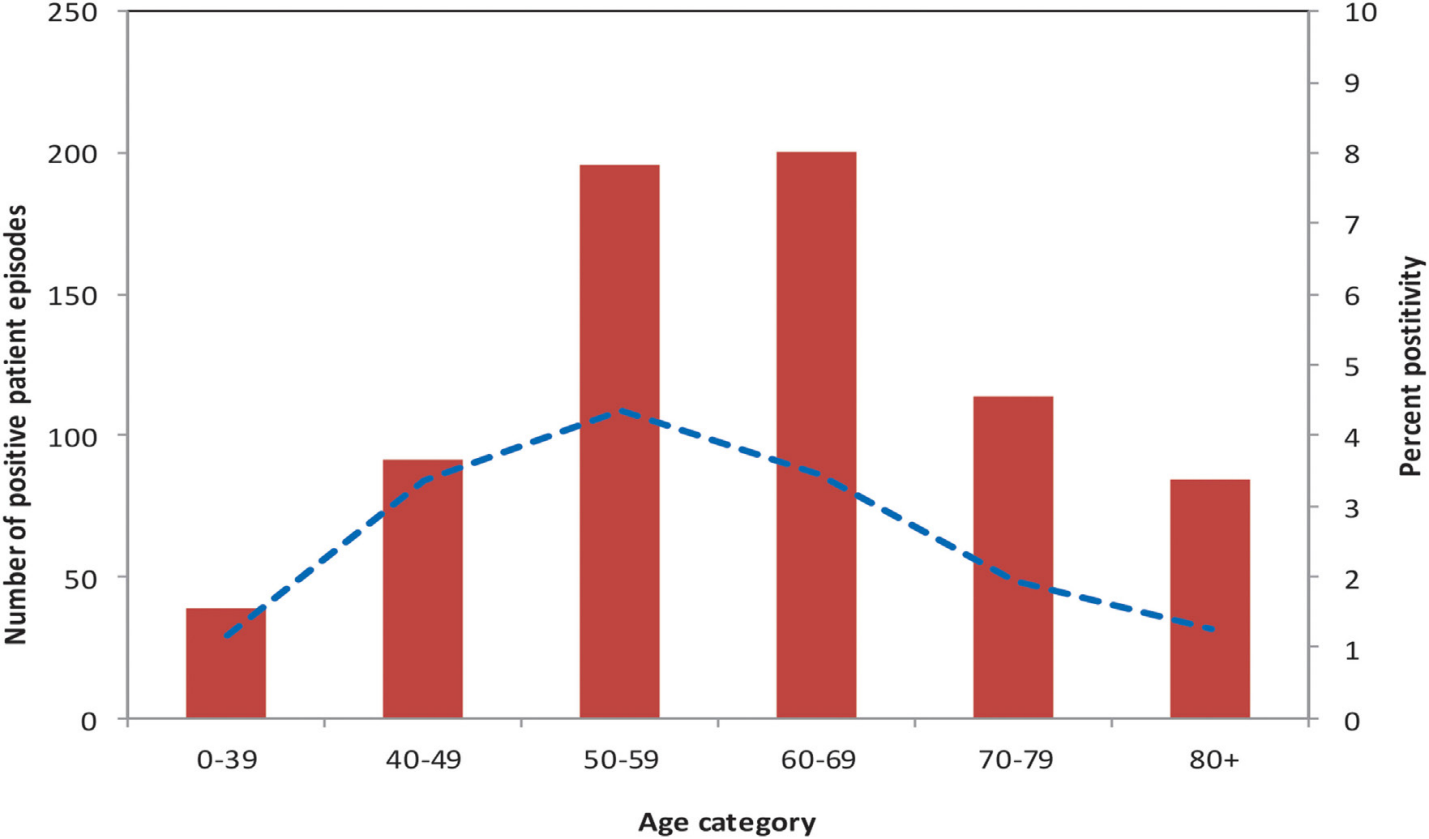
**Number of *Legionella* cases, and precent of patients test-positive, by age group, PHOL, 1st January, 2010 to 30th April, 2014**.

The mean age of patients tested for *Legionella* was 63 years, with a range of 2 days to 102 years. There was a statistically significant difference in age between individuals testing positive for *Legionella* (cases) and those testing negative (controls) (*p*-value < 0.001). Compared to controls, cases were more likely to be in the 40 to 69 year age group. The age group with the highest proportion of individuals testing positive for *Legionella* (4.3%) was the 50 to 59 year group (Figure 2). Of patients tested for *Legionella*, 15,625 (54.4%) were males. Five hundred and five cases (69.9%) were males compared with 15,120 (54.1%) controls. Cases were more likely than controls to be males (*p*-value < 0.001).

### Testing Characteristics

A range of 1–17 specimens were submitted per patient for *Legionella* testing during the study period; 27,561 (95.2%) patients had specimens submitted for a single episode, and 22,105 (76%) patients had only one specimen submitted. Of patients with specimens submitted for multiple episodes, none tested positive for *Legionella* for more than a single episode. The maximum number of episodes for which any patient had specimens submitted was six, and these were related to a single patient who was immunocompromised. *Legionella* was not identified in any specimens tested from this patient.

Of the patients who had more than one *Legionella*-positive specimen (*n* = 149), the mean number of days that any patient remained *Legionella*-positive was 5.1 days with a range of 0–67 days and SD of 11.8 days. Of all cases, 576 (79.5%) had only one positive specimen. One patient tested positive for *Legionella* for 67 days. This patient had nine specimens tested out of which eight were positive for *L. pneumophila* serogroup 1. This also represented the highest number of positive specimens for any patient. Specimen sources submitted from this patient included urine, sputum, and BAL; the patient continued to test positive by culture and PCR for 45 days from the time of the first positive specimen and tested positive by UAT until 67 days after the first positive test result.

### *Legionella* Serogroups

Of all *Legionella* species identified during our study, 700 (96.6%) were *L. pneumophila*, and 25 (3.4%) were *L. non-pneumophila*. *L. pneumophila* serogroup 1 represented 680 (93.8%) of all *Legionella* identified to the species or higher level by PHOL; all *Legionella* species identified at PHOL are documented in Table 1.

**Table 1:** ***Legionella* species and serogroups identified among 725 *Legionella* cases by PHOL, January 2010 to April 2014**.

*Legionella* species	*Legionella* serogroup (sg)	Counts	Percentage
*L. pneumophila*	*L. pneumophila* not serotyped^a^	15	2.1
	*L. pneumophila* sg1^b^	680	93.8
	*L. pneumophila* sg2	1	0.1
	*L. pneumophila* sg3	2	0.3
	*L. pneumophila* sg6	1	0.1
	*L. pneumophila* sg9	1	0.1
Subtotal of *L. pneumophila*		700	96.6
*L. non-pneumophila*	*L. longbeachae1*	1	0.1
	*L. maceachernii*	1	0.1
	*L. micdadei*	2	0.3
	*L. non-pneumophila* not serotyped	21	2.9
Subtotal of *L. non-pneumophila*		25	3.4
Overall		725	100.0

*^a^Not serotyped meaning that *Legionella pneumophila* was identified by PCR alone (*n* = 13), or an attempt to serotype was made but the serotype could not be determined (*n* = 2)*.

*^b^The majority (523; 72.4%) of patients with *L. pneumophila* sg1 infection were tested by UAT only*.

*sg, serogroup*.

### Test Method Comparison

When comparing UAT to culture, *Legionella* culture was considered the gold standard as it can detect all *Legionella* species. Overall, 795 patients were tested by UAT and *Legionella* culture during the same episode (Table 2). Of these, 67 (8.4%) patients were *Legionella-*positive by both UAT and culture. Ten patients had a positive *Legionella* result by culture alone and 38 patients by UAT alone. Sensitivity of UAT compared to culture was 87%, specificity was 94.7%, positive predictive value (PPV) was 63.8%, and negative predictive value (NPV) was 98.5% (Table 3). *Legionella* was not detected by UAT in 10 (12%) patients for whom culture was positive. Of these 10 patients, 6 were *Legionella* culture-positive for *L. pneumophila* serogroup 1 and the other patients were positive for *L. pneumophila* serogroup 6 (*n* = 1), *L. micdadei* (*n* = 1), *L. longbeachae* (*n* = 1), and *L. maceachernii* (*n* = 1). Conversely, culture did not detect *L. pneumophila* serogroup 1 in 38 (36%) patients for whom UAT had a positive test result. The overall estimates for UAT were compared to culture only for the period before implementation of PCR. All estimates were not significantly different except specificity of UAT, which was slightly higher prior to the implementation of PCR (97.7 vs. 94.7%, *p*-value < 0.05).

**Table 2:** **Comparing *Legionella* test methods, PHOL 1st January, 2010 to 30th April, 2014**.

	Culture			PCR^a^		
Binax	Positive	Negative	Total	Positive	Negative	Total
Positive	67	38	105	56	16	72
Negative	10	680	690	19	961	980
Total	77	718	795	75	977	1,052

*^a^Four patients with indeterminate results by PCR were excluded from the analyses*.

**Table 3:** **Comparison of performance indicators for *Legionella* UAT, culture and PCR, PHOL, January 1, 2010 to April 30, 2014£**.

Performance indicators		Binax vs. culture	Binax vs. PCR	Culture vs. PCR
TP/(TP + FN)	Sensitivity	87.0 (76.9–93.2)	74.7 (63.1–83.7)	45.2 (37–53)
TN/(TN + FP)	Specificity	94.7 (76.9–93.2)	98.3 (97.2–99.0)	NA
TP/(TP + FP)	PPV	63.8 (53.7–72.7)	77.7 (66.1–86.3)	NA
TN/(TN + FN)	NPV	98.5 (97.2–99.2)	98.1 (96.9–98.7)	NA

*TP, true positive; TN, true negative; FN, false-negative; FP, false-positive; NA, not applicable*.

Comparing *Legionella* UAT to PCR, we considered PCR to be the reference test based on previously published results as well as its greater ability to detect all *Legionella* species (11). Overall, there were 1,052 patients who had both UAT and PCR performed (Table 2). Fifty-six specimens were positive for *Legionella* species by both UAT and PCR, 19 patients were positive by PCR alone, and 16 patients were positive by UAT alone. UAT did not identify *Legionella* for 19 (25%) patients for whom PCR had a positive result. Of these 19 patients, *L. pneumophila* was detected in 9 patients and *L. non-pneumophila* was detected in 10. Of those with *L. pneumophila*, three had results confirmed by culture for *L. pneumophila* serogroup 1 (*n* = 2) and *L. pneumophila* serogroup 6 (*n* = 1); the rest did not have serogroup testing performed. Conversely, 16 (22.2%) patients in whom *L. pneumophila* serogroup 1 was confirmed by UAT were negative by PCR. Sensitivity of UAT compared to PCR was 74.7%, specificity was 98.3%, PPV was 77.7%, and NPV was 98.1% (Table 3).

We were unable to fully compare and report all parameters for culture and PCR since subsequent to 28th May, 2012, culture was only used for patients testing positive for *Legionella* by PCR. Of 146 patients who had a *Legionella-*positive result by PCR, only 66 (45.2%) also had a positive result by culture (Table 3). Specimens from 80 patients did not grow in culture, and of these, 23 were identified by PCR as *L. non-pneumophila*, and 57 were *L. pneumophila* not serotyped.

### Specimen Source Comparison

Percent positivity of sputum and BAL were compared to understand the utility of sputum as an appropriate specimen source to detect *Legionella*. Out of 625 sputum specimens tested, 64 (10.2%) were positive for *Legionella*. Out of 9,275 BAL specimens tested, 215 (2.3%) were positive for *Legionella*. Overall, percent positivity in sputum was higher compared to BAL (*p*-value < 0.05). When we compared sputum and BAL by test method, 30/297 (10.1%) sputum specimens and 106/4,697 (2.3%) BAL specimens were positive for *Legionella* by culture. Testing by *Legionella* PCR identified *Legionella* in 34/326 (10.4%) sputum specimens and in 109/4,568 (2.4%) BAL specimens. Percent positivity for *Legionella* was higher in sputum as opposed to BAL regardless of test method (*p*-value < 0.05). Culture results were then analyzed only for the period prior to implementation of PCR since, subsequent to its introduction, culture was only used for PCR-positive specimens. Percent positivity was reduced for both sputum and BAL; specifically of 254 sputum specimens tested by *Legionella* culture before 28th May, 2012, 9 (3.5%) were positive for *Legionella*, and out of 4,447 BAL specimens tested by culture, 55 (1.2%) were positive for *Legionella*. Positivity was higher in sputum as opposed to BAL; however, the difference was smaller when compared with the entire study period.

Further, we compared test results of sputum and BAL controlling for test method and patient episode. For this analysis, we compared only those specimens that were tested from the same patient within the same episode and within a 14-day lag period. This time frame was selected to avoid bias related to delays in testing, which may affect results. Twenty-two patients had both sputum and BAL specimens tested by culture and 39 patients had sputum and BAL tested by PCR. Sensitivity for culture was the same using either sputum or BAL (13.6%) as the source (Fisher Exact *p*-value = 1.0). Sensitivity for PCR was 10.3% for sputum and 12.8% for BAL; results were not significantly different (Fisher Exact *p*-value = 1.0). When we checked culture results just for the period prior to introduction of PCR, sensitivity of sputum vs. BAL were not significantly different (Fisher Exact *p*-value = 1.0).

In addition to sputum and BAL, *Legionella* was also identified in 2/44 (4.5%) aspirate (exact source not documented on test requisition), 2/78 (2.6%) pleural fluid, and 3/185 (1.6%) specimens with an unspecified source. *Legionella* was not identified in autopsy (*n* = 15), blood (*n* = 2), lung tissue (*n* = 361), nasopharyngeal swab (*n* = 22), or tracheal tissue specimens (*n* = 18).

## Discussion

In this study, we describe *Legionella* testing at PHOL. *Legionella* was identified in approximately 3% of patients and percent positivity for each test method (UAT or culture or PCR) was also approximately 3%. As reported elsewhere, *Legionella* detection peaked in late summer (14) with *Legionella* most commonly identified in older males (4). Seventy-six percent of patients tested for *Legionella* species had only one specimen submitted and almost 70% of the patients were tested by UAT alone. As reported previously, *L. pneumophila* serogroup 1 was the most common serotype identified in Ontario (1); less than 4% of positive *Legionella* results were *L. non-pneumophila*. However, we may have overestimated the prevalence of *L. pneumophila* serogroup 1 as UAT alone was the predominant testing method used at PHOL, because many patients only have urine specimens submitted for *Legionella* testing.

A higher number of BAL than sputum specimens were submitted at PHOL for *Legionella* testing. Historically, BAL has been considered to be the optimal respiratory specimen for identification of *Legionella*, likely because less than one-half of patients infected with *Legionella* produce sputum (2). Percent positivity in sputum was higher than BAL for both culture and PCR. However, when we compared the performance of the two sources, controlling for patient episode (i.e., same patient and within the same episode), positivity of sputum and BAL were not significantly different (Fisher exact *p*-value = 1). This finding contrasts results of a previous study in which positivity of culture results were reported to be higher for BAL than sputum (15); however, that study had a very small number of patients and sputum, and BAL were not collected from the same patient within the same disease episode. In a systematic review, subgroup analysis by specimen type found similar sensitivity and specificity for sputum and BAL specimens tested by PCR (11).

A key added benefit to the use of sputum is that a sample can easily be obtained from patients, and it is a non-invasive procedure.

When comparing UAT to culture, UAT demonstrated high sensitivity (87%) and very high specificity (97.7%). Binax detected *L. pneumophila* serogroup 1 in 38 patients for whom culture had a negative result. This may reflect that culture has a lower reported sensitivity (10). However, some of the positive results by UAT alone may indicate false-positive results. False-positive results by urine antigenic testing have been reported previously and have been attributed to serum sickness or the presence of rheumatoid factor (16). Testing by *Legionella* PCR could have differentiated this; however, we were unable to perform further testing due to the retrospective nature of this study.

In addition, UAT indicated a negative test result for 10 specimens, which culture reported as *Legionella* positive. The inability of UAT to detect species/serogroups other than *L. pneumophila* serogroup1 may explain part of the discrepancy. However, when we investigated the reasons for discordance, only 6 out of 10 were identified as *L. pneumophila* serogroup 1 species. False negative results by UAT might be due to the critical need of immediate testing of urine specimens as prolonged time prior to testing may result in the degrading of urine antigen (17). In addition, false negative UAT results may be related to lower sensitivity of UAT in milder *Legionella* infections or to the need for higher concentration of urine samples when performing UAT (18). At PHOL, we do not routinely concentrate urine specimens before testing. Specificity of UAT in comparison to culture dropped after PCR introduction. This was due to the fact that culture was used only on PCR-positive specimens – a subset of patients who had true *Legionella* infection were missed by culture but were picked up by UAT, falsely decreasing the specificity of urine when using culture as the reference method. The same factor contributed to the poor PPV (63.8%) for UAT.

When comparing *Legionella* UAT to PCR, considering PCR as the reference method, UAT demonstrated a moderate sensitivity (74.7%) and very high specificity (98.3%). UAT detected 16 *L. pneumophlia* serogroup 1 positive specimens that were not detected by PCR. This may be attributed to false-positive UAT results (explained above) or false-negative PCR results due to PCR inhibition, primer and/or probe mismatch, the presence of *Legionella* target in quantities below the limit of detection or improper specimen collection and handling (19). UAT did not detect 19 *Legionella*-positive specimens detected by PCR and half of them were *L. pneumophila* serogroup 1. While false-positive PCR results may occur as a result of specimen contamination with *Acinetobacter* spp. or *Gemella* spp. (18), it is more likely that the discordant result was the result of higher PCR sensitivity. At PHOL, we also identified that *Legionella* PCR gave a false-positive result in at least three patients due to cross-reaction with *Stenotrophomonas maltophilia* by the *Legionella* species target but not the *L. pneumophila*.

*Legionella* culture results could not be compared to PCR results due to the change in testing algorithm, whereby culture was only set up if respiratory specimens were PCR-positive. However, of all specimens positive for *Legionella* by PCR, only 45% were successfully grown in culture. Sensitivity of culture compared with PCR (45%) was much lower than the sensitivity of UAT to PCR (74.7%). The low sensitivity, longer turnaround time, and technical expertise required to isolate *Legionella* spp. in culture resulted in culture not being the method of choice for detecting *Legionella* (20). However, culture is still useful for isolating the bacteria to facilitate the linkage between clinical to environmental specimens by molecular typing, usually SBT.

Finally, we were able to report on prolonged *Legionella* detection from a single patient. The patient tested positive for 67 days after the first positive specimen, with the last positive specimen source being urine, tested by UAT. We were able to do so because the patient had nine specimens submitted for testing. Detection of *Legionella* antigen in the urine has been reported for as long as 300 days (21). Prolonged detection from urine complicates interpretation of positive results with respect to the timing of antibiotic treatment.

This study has some limitations. *Legionella* cases reported here may not represent all *Legionella* cases in Ontario for two main reasons: many cases with PF are not detected and thus not reported (22). In addition, the Alere Binax UAT is not approved for testing PF patients, and its test characteristics in PF have not been well characterized. As well, patients tested by UAT who are infected with species other than *L. pneumophila* serogroup 1 will have unrecognized infection. Test methods were not used consistently during the study period, which prevented us from conducting a comprehensive comparison between different test methods. The change in testing algorithm after implementation of PCR prevented us from being able to compare culture and PCR test methods, as culture was only performed on PCR-positive specimens post PCR implementation, for the purpose of facilitating molecular typing by SBT when indicated. It also prevented us from comparing UAT, culture, and PCR results among all patients who were tested by all three methods.

Since clinical information was not reported consistently on laboratory requisitions, we could not clinically describe patients from whom specimens were collected or comment on their disease severity or trajectory. Last, comprehensive testing to determine the cause of clinical illness may result in an unrepresentative sample or sampling biases, with sicker patients more likely to be tested for *Legionella* using multiple test methods.

## Conclusion

In summary, all test methods have inherent weaknesses for the identification of *Legionella*; therefore, more than one testing method should be used. Obtaining a single specimen type from patients with pneumonia limits the ability to diagnose *Legionella*, particularly when urine is the specimen type submitted. Additionally, detection of *Legionella* using urine alone does not facilitate identification of linkages between clinical isolates and potential environmental exposures, which is achieved by molecular typing, specifically SBT. Given ease of collection, and its similar sensitivity to BAL, clinicians are encouraged to submit sputum in addition to urine when BAL submission is not practical, from patients being tested for *Legionella*.

## Author Contributions

Conceived, designed the research, analyzed the data, and wrote the manuscript: AP, A-LW, and JBG.

## Conflict of Interest Statement

The authors declare that the research was conducted in the absence of any commercial or financial relationships that could be construed as a potential conflict of interest.
